# In Vitro Activity of a Novel Siderophore-Cephalosporin, GT-1 and Serine-Type β-Lactamase Inhibitor, GT-055, against *Escherichia coli*, *Klebsiella pneumoniae* and *Acinetobacter* spp. Panel Strains

**DOI:** 10.3390/antibiotics9050267

**Published:** 2020-05-20

**Authors:** Le Phuong Nguyen, Naina Adren Pinto, Thao Nguyen Vu, Hyunsook Lee, Young Lag Cho, Jung-Hyun Byun, Roshan D’Souza, Dongeun Yong

**Affiliations:** 1 Department of Laboratory Medicine and Research Institute of Bacterial Resistance, College of Medicine, Yonsei University, Seoul 03722, Korea; luongphekidz07@gmail.com (L.P.N.); naina.pinto@gmail.com (N.A.P.); vuthaonguyen1992@gmail.com (T.N.V.); snoopycat78@gmail.com (H.L.); 2 Brain Korea 21 PLUS Project for Medical Science, Yonsei University, Seoul 03722, Korea; 3 Legochem Biosciences, Daejeon 34302, Korea; young@legochembio.com; 4 Department of Laboratory Medicine, Gyeongsang National University College of Medicine, Gyeongsang National University Hospital, Jinju 52727, Korea; microbyun@gmail.com; 5 J. Craig Venter Institute, Rockville, MD 20850, USA

**Keywords:** GT-1, GT-055, siderophore-cephalosporin, β-lactamase inhibitor

## Abstract

This study investigates GT-1 (also known as LCB10-0200), a novel-siderophore cephalosporin, inhibited multidrug-resistant (MDR) Gram-negative pathogen, via a Trojan horse strategy exploiting iron-uptake systems. We investigated GT-1 activity and the role of siderophore uptake systems, and the combination of GT-1 and a non-β-lactam β-lactamase inhibitor (BLI) of diazabicyclooctane, GT-055, (also referred to as LCB18-055) against molecularly characterised resistant *Escherichia coli*, *Klebsiella pneumoniae* and *Acinetobacter* spp. isolates. GT-1 and GT-1/GT-055 were tested in vitro against comparators among three different characterised panel strain sets. Bacterial resistome and siderophore uptake systems were characterised to elucidate the genetic basis for GT-1 minimum inhibitory concentrations (MICs). GT-1 exhibited in vitro activity (≤2 μg/mL MICs) against many MDR isolates, including extended-spectrum β-lactamase (ESBL)- and carbapenemase-producing *E. coli* and *K. pneumoniae* and oxacillinase (OXA)-producing *Acinetobacter* spp. GT-1 also inhibited strains with mutated siderophore transporters and porins. Although BLI GT-055 exhibited intrinsic activity (MIC 2–8 μg/mL) against most *E. coli* and *K. pneumoniae* isolates, GT-055 enhanced the activity of GT-1 against many GT-1–resistant strains. Compared with CAZ-AVI, GT-1/GT-055 exhibited lower MICs against *E. coli* and *K. pneumoniae* isolates. GT-1 demonstrated potent in vitro activity against clinical panel strains of *E. coli*, *K. pneumoniae* and *Acinetobacter* spp. GT-055 enhanced the in vitro activity of GT-1 against many GT-1–resistant strains.

## 1. Introduction

The World Health Organisation has prioritised third-generation cephalosporin and carbapenem-resistant Enterobacteriaceae and *Acinetobacter baumannii* as critical pathogens for research and development of new antibiotic drugs [1]. More importantly, the emergence of strains co-harbouring carbapnemases, such as *bla*_KPC-2_ or *bla*_NDM-9_, and colistin resistance gene, such as *mcr-1*, have made treatment more challenging [2,3]. This has highlighted the importance of developing new antibiotics to address the problem of antibiotic resistance. However, in contrast to the rapid and continuous escalation in the emergence of multidrug-resistant (MDR) Gram-negative bacteria and the diminishing efficacy of the current antibiotic arsenal, there has been a lag in investment in new research and drug development [1]. A bottleneck in current approaches for designing novel antibiotics is the limited number of drug targets, such as components of nucleic acid synthesis and cell wall assembly, or bacterial ribosomes [4].

Since the first antibiotics were discovered, Gram-negative bacteria have developed various resistance mechanisms, such as hydrolysis or modification of antibiotics, reducing antibiotic uptake via loss of porins, reducing the affinity of antibiotic-target binding and increasing the expression of efflux pumps [5,6]. Moreover, the outer membrane barrier is a crucial resistance determinant in Gram-negative bacteria, as reduced permeability of the cell wall potentiates other antibiotic resistance mechanisms.

**Figure 1:**
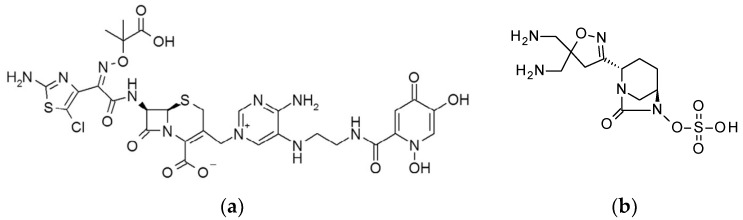
Structures of GT-1 and GT-055. (**a**) Chemical structure of GT-1 (previously known as LCB10-0200). (**b**) Chemical structure of GT-055 (previously known as LCB18-055).

The novel siderophore-cephalosporin GT-1 (previously known as LCB10-0200) is in development by LegoChem Biosciences (Daejeon, Korea) and Geom Therapeutics (San Francisco, CA, USA). This compound is a conjugate of a novel siderophore-dihydroxypyridone and a modified aminothiazolylglycyl cephalosporin and exploits bacterial iron-uptake systems to enhance entry into Gram-negative pathogens using a “Trojan-horse” strategy [7] (Figure 1a). First, this approach opens a more efficient route for the antibiotic to enter the bacterial periplasm via siderophore-uptake systems. Second, the conjugated structure resists the hydrolytic activity of many current extended-spectrum β-lactamases and carbapenemases. As a result, GT-1 exhibits activity against many MDR pathogens.

One well-described strategy to enhance the activity of β-lactam antimicrobials in the presence of β-lactamases is to combine them with β-lactam inhibitors (BLIs) [8]. In the last 5 years, new generations of BLIs have been approved for clinical use, including vaborbactam (cyclic boronic acid BLI) and avibactam (diazabicyclooctane BLI) [9,10]. GT-055 (also referred to as LCB18-055), in development by LegoChem Biosciences and Geom Therapeutics, is a novel non-β-lactam BLI of the diazabicyclooctane class [11] (Figure 1b).

In a previous study, Oh et al. characterised the in vitro activity of GT-1, focusing primarily on *Pseudomonas aeruginosa* [7]. Only 17 *P. aeruginosa* strains with identified β-lactamases (OXA-2, OXA-10, OXA-17, VIM and IMP) were investigated. Even though the in vitro activity of GT-1 was also determined in other species, such as *Escherichia coli*, *Klebsiella pneumoniae* and *A. baumannii*, the resistome profile of the tested strains has not been described. In this study, the in vitro activity of GT-1 and other antibiotics was investigated against panels of characterised isolates of *E. coli*, *K. pneumoniae* and *Acinetobacter* spp., which exhibit a variety of β-lactam resistance mechanisms. Additionally, this is the first study to examine the synergistic combination of GT-1 and GT-055 and compare its potency to other combinations, such as azithromycin/avibactam and ceftazidime/avibactam. Until now, there has been no systematic investigation of the correlation between GT-1 potency and the different iron uptake systems of *E. coli*, *K. pneumoniae* and *Acinetobacter* spp. clinical isolates. Moreover, there has been no report describing other mechanisms that can contribute to resistance to GT-1, such as GT-1 hydrolysis or porin loss. Thus, this study was undertaken to elucidate the genomic characteristics of the panel strains and associated GT-1 minimal inhibitory concentrations (MICs).

## 2. Results

### 2.1. In Vitro Activity of GT-1 and GT-1/GT-055 against E. coli Panel Strains

GT-1 activity was tested against isolates producing a wide range of β-lactamases. The strains were isolated from different specimen types, including stool (9 isolates), blood (1 isolate), pus (1 isolate), respiratory tract (1 isolate) and peritoneal fluid (1 isolate). The β-lactamase producers present in these diverse specimens included strains producing non-extended-spectrum β-lactamase (ESBLs) (TEM-1B, TEM-1C, SHV-11, OXA-1, OXA-10), ESBLs (CTX-M-14, CTX-M-15, CTX-M-27, CTX-M-55, CTX-M-65), AmpC (ACT-2, CMY-2) and carbapenemases (KPC-2, OXA-48) (Table 1). The strains were classified into six categories: non-ESBL-producing (YMC2016/07/N348), ESBL-producing (YMC2016/06/N138, YMC2017/02/N55), ESBL- and AmpC β-lactamase-producing (YMC2017/02/N19), AmpC β-lactamase- and carbapenemase-producing (YMC2017/07/B11634), ESBL- and carbapenemase-producing (YMC2016/02/N176, YMC2016/06/N255, YMC2017/04/N120, YMC2017/06/P238) and carbapenemase-producing (YMC2016/01/N95, YMC2016/01/C905, YMC2016/04/R3267, YMC2016/10/N189) (Table 1). 

The GT-1 MICs ranged from ≤0.12 to 1 μg/mL against *E. coli* MDR isolates producing KPC- or OXA-carbapenemases, ESBLs CTX-M-14 or CTX-M-55 and CMY-2 AmpC β-lactamase. These MICs were lower in comparison to those for carbapenem, which ranged from 0.25 to 16 μg/mL, or in comparison to MICs for ceftazidime, which ranged from 1 to 64 μg/mL. Against CTX-M-negative but carbapenemase-producing *E. coli*, GT-1 MICs were ≤0.25 μg/mL, whereas carbapenem MICs ranged from 0.5 to 16 μg/mL. ESBL-producing *E. coli* strains (CTX-M-15, CTX-M-27, CTX-M-65) and strains overexpressing ACT-2 exhibited GT-1 MICs of 4–32 μg/mL.

In combination with the β-lactamase inhibitor GT-055 (at 4 μg/mL), GT-1/GT-055 MICs for all strains decreased to ≤0.12 μg/mL, similar to or lower than the MICs for the combination of CAZ-AVI (Table 1). GT-055 alone exhibited activity against the *E. coli* isolates with MICs ranging from 2 to 8 μg/mL.

The siderophore uptake system and *ompC* and *ompF* integrity were investigated to evaluate the contribution of the siderophore transporters and porin loss to the MIC of GT-1 (Table 2). There were no deletions or early terminations in the *tonB*, *exbB*, *exbD*, *fiu*, or *fepA* genes in any of the isolates. The GT-1 MIC was ≤0.5 μg/mL with the triple deletion of *fecA*, *fhuA* and *iroN* in strain YMC2016/06/N138 or the deletion of *fecA*, *iutA*, *fyuA* and *iroN* in strains YMC2017/07/B11634, YMC2016/01/N95, YMC2016/10/N189, as well as the deletion of *iutA*, *fhuA* and *iroN* in strain YMC2016/06/N255. Furthermore, the deletion of both *ompC* and *ompF* in strain YMC2016/06/N138 and deletion of *ompF* in strain YMC2016/10/N189 did not appear to be associated with increases in GT-1 MIC. Although strain YMC2016/02/N176 exhibited a high GT-1 MIC of 8 μg/mL, there was one observed deletion of *fyuA* without porin loss. In this case, the β-lactamases present were likely responsible for the high MIC, as the combination with GT-055 reduced the MIC to ≤0.12 μg/mL.

### 2.2. In Vitro Activity of GT-1 and GT-1/GT-055 against K. pneumoniae Panel Strains

The activity of GT-1 was tested against *K. pneumoniae* isolates that produce a broad range of β-lactamases, including four ESBL-producing strains, four ESBL- and AmpC β-lactamase– co-producing strains, three ESBL- and carbapenemase-co-producing strains, and one carbapenemase-producing strain, with a variety of multilocus sequence types (MLSTs) (Table 1). The β-lactamase producers present in the diverse panel of strains included producers of non-ESBLs (OXA-1, OXA-2, OXA-4, OXA-9, LEN-7, LEN-12, TEM-1A, TEM-1B, SHV-11), ESBLs (SHV-12, SHV-83, CTX-M-15), AmpC (DHA-1), and carbapenemases (KPC-2, IMP-1). The isolates were collected from a variety of sources, including stool (two isolates), blood (8 isolates), pus (1 isolate), respiratory tract (two isolates), and peritoneal fluid (one isolate).

GT-1 MICs ranged from 0.5 to 1 μg/mL against KPC-2- and IMP-1-producing *K. pneumoniae*. These MICs were ≥16-fold lower compared with carbapenems, for which the MICs ranged from 16 to 64 μg/mL. GT-1 MICs against the ESBL-producing strains SHV-12, SHV-83 and CTX-M-15 ranged from 0.25 to 4 μg/mL, 8- to 64-fold lower than the ceftazidime MICs and 2- to 8-fold lower than the carbapenem MICs. Among five isolates expressing the DHA-1 β-lactamase, four isolates exhibited high GT-1 MICs (4–64 μg/mL). Although strain YMC2016/04/N62 was resistant to colistin (MIC = 32 μg/mL), it exhibited a GT-1 MIC of 0.5 μg/mL. To directly assess the activity of GT-1 in the presence of DHA-1 β-lactamase, *bla*_DHA-1_ was cloned into ZpUC19 and transformed into *E. coli* DH5α, which resulted in a 16-fold increase in the GT-1 MIC compared to the control carrying the empty ZpUC19 vector (Table 3). However, in combination with the β-lactamase inhibitor GT-055, the GT-1/GT-055 MICs decreased to ≤0.12 μg/mL, except for strains YMC2010/10/P347 and YMC2012/08/C631. In YMC2010/10/P347, the GT-1 MIC decreased 4-fold to 16 μg/mL when combined with GT-055, which was similar to the MIC for GT-055 alone. No change in GT-1 MIC (0.5 μg/mL) was observed against strain YMC2012/08/C631 (IMP-1) in combination with GT-055.

The siderophore uptake systems and integrity of *ompK35* and *ompK36* were also investigated to elucidate the role of the siderophore transporters and porin loss on GT-1 MICs against *K. pneumoniae* (Table 2). No deletion or early termination of *tonB* or *exbD* was observed, but *iroN* was absent in all of the isolates. The deletion of *exbB* was observed in strain YMC2011/8/B10311, but the GT-1 MIC was low at 0.5 μg/mL. The GT-1 MIC was ≤1 μg/mL in strain YMC2011/07/B7207, in which *fecA*, *fiu* and *fyuA* were deleted; in strain YMC2013/7/B3993 in the absence of *fepD*, *fiu*, *cirA* and *fyuA*, in strain YMC2011/7/B774 with the deletion of *fecA*, *fiu*, *fhuA* and *fyuA*, and in strain YMC2012/8/C631 with the deletion of *fecA*, *fiu*, *cirA*, *fhuA* and *fyuA*. Additionally, the single deletion of *ompK35* in strain YMC2013/7/B3993 and double deletion of *ompK35* and *ompK36* in strain YMC2010/10/R2142 did not result in high GT-1 MICs. This suggested that mutations in the siderophore transporter genes *fecA*, *fiu*, *cirA*, *fhuA*, or *fyuA* and the porin genes *ompK35* or *ompK36* do not markedly affect GT-1 MICs. Early termination of *fiu* in strain YMC2011/7/B36 and deletion of *fecA* in strain YMC2011/11/B7578 were associated with GT-1 MICs of 16 and 64 μg/mL, respectively. However, similar mutations were also observed in strains exhibiting low GT-1 MICs, so the importance, if any, of these mutations in determining GT-1 MICs in strains YMC2011/7/B36 and YMC2011/11/B7578 remains unclear. Although there was only one deletion of *iroN* in the siderophore uptake system in strain YMC2010/10/P347, this strain exhibited a high GT-1 MIC of 64 μg/mL. The low MIC for the combination of GT-1/GT-055 with this isolate (relative to GT-1 alone or GT-055 alone) was consistent with the high GT-1 MICs reflecting the activity of β-lactamases inhibited by GT-055.

### 2.3. In Vitro Activity of GT-1 and GT-1/GT-055 against Acinetobacter spp. Panel Strains

The antimicrobial activity of GT-1 and GT-055 was examined against *Acinetobacter* spp. isolates exhibiting various β-lactam resistance mechanisms, including one narrow-spectrum oxacillinase–producing strain, three ESBL- and AmpC-co-producing strains, two AmpC- and carbapenemase-co-producing strains and five ESBL-, AmpC- and carbapenemase-co-producing strains. The broad range of β-lactamase producers represented among the panel strains included non-ESBL (CARB-8, TEM-1D), ESBL (PER-1, OXA-66), AmpC (ADC-22, ADC-25, ADC-30, ADC-31, ADC-77), and carbapenemase (OXA-23, OXA-82, OXA-120, OXA-213, OXA-421, OXA-499, OXA-506, SIM-1) producers (Table 1). The samples were isolated from different sources, including stool (6 isolates), blood (2 isolates), peritoneal fluid (2 isolates), and catheter tip (1 isolate).

GT-1 MICs ranged from 0.5 to 2 μg/mL among isolates producing OXA-ESBL (OXA-66), OXA-carbapenemases (OXA-23, OXA-82, OXA-120, OXA-213, OXA-421, OXA-499), and ADC-type (ADC-25, ADC-30, ADC-77) AmpC β-lactamases. A reduction of >64- to 256-fold in GT-1 MICs was observed when compared to ceftazidime (MICs 4 to >256 μg/mL), and a 16- to 64-fold reduction was observed when compared to carbapenem (MICs 0.5 to 32 μg/mL). Nonetheless, strains producing PER-1 exhibited high GT-1 MICs, ranging from 16 to 256 μg/mL, and *E. coli* DH5α encoding ZpUC19::*bla*_PER-1_ exhibited a 1024-fold increase in GT-1 MIC in comparison with *E. coli* DH5α harbouring the empty vector (Table 3). The increase in GT-1 MIC supports the hypothesis that GT-1 is susceptible to PER-1 β-lactamase.

GT-1, in combination with GT-055, decreased the MICs for several ESBL-AmpC- and ESBL-AmpC carbapenemase-producing isolates. In particular, the GT-1 MIC decreased by 32-fold in strain YMC2003/01/R306, which produces PER-1 and ADC-25. In addition, a 32-fold reduction in GT-1 MIC from 256 to 8 μg/mL was observed in strain YMC2003/05/C86, which produces PER-1, ADC-31 and OXA-82. However, the combination of GT-055 and GT-1 was not as significant as it was against *E. coli* and *K. pneumoniae*, with only 2 of 11 strains exhibiting a more than 4-fold reduction in MIC (Table 1). GT-1/GT-055 MICs were equal to or lower than CAZ-AVI or AZT-AVI for all isolates. Specifically, CAZ-AVI MICs ranged from 4 to >256 μg/mL, and AZT-AVI MICs ranged from 8 to 128 μg/mL, whereas GT-1/GT-055 MICs ranged from 1 to 128 μg/mL. In addition, GT-055 exhibited no detectable intrinsic activity against any of the *Acinetobacter* spp. isolates (MIC > 256 μg/mL).

The porin and siderophore uptake systems and their correlation with GT-1 MICs were also investigated (Table 2). Only one *tonB* system is present in *E. coli* and *K. pneumoniae*. Whereas, in *Acinetobacter* spp., there are three different *tonB* systems, including *tonB*, *tonB2* and *tonB3*. However, the *tonB3* system was recognised as the main siderophore uptake system [12], and it was well conserved among the strains we tested. Only 4/11 strains (YMC2012/07/R3167, YMC2011/02/C582, YMC2003/05/C86, YMC2013/03/R2081) harboured intact *tonB*, *exbB* and *exbD* genes. Only 2/11 strains (YMC2013/03/R2081 and YMC2009/02/B2968) had early termination of *exbD3*, with GT-1 MICs of 16 and 1 μg/mL, respectively. The low GT-1 MIC against the *exbD3* mutant strain YMC2009/02/B2968 demonstrated that the deletion of *exbD3* has no effect on GT-1 MIC. In addition, 10/11 and 9/11 strains had a deletion or early termination in *bauA* and *piuA*, respectively. The absence or presence of *pirA*, *fhuA* and *bfnH* did not correlate with high GT-1 MICs against *Acinetobacter* spp. Moreover, no deletions of *oprD*, *carD*, or *33_36 kDa* were observed in the strains exhibiting a high GT-1 MIC (YMC2003/01/R306, YMC2011/02/C582 and YMC2003/05/C86). This suggested that the high GT-1 MICs against *Acinetobacter* spp. were not caused by the loss of porins.

## 3. Discussion

Resistance among Gram-negative species to existing classes of antimicrobials, including carbapenems, has limited treatment options for clinicians. In some cases, the only effective option is colistin, a drug once abandoned due to its serious side effect profile. However, the effectiveness of colistin has decreased as a result of the rapid spread of *mcr*-like genes [13]. The next generation of antibiotics should include new agents that would impede the development of antibiotic resistance in MDR bacteria. Siderophore-antibiotic conjugants may partially fulfil this requirement. Because ferric ion is indispensable for bacterial growth and virulence [14,15], any mutations in siderophore uptake systems during antibiotic treatment could lead to the loss of bacterial virulence, thus ultimately reducing pathogenicity in host cells. In addition, siderophore uptake may involve more than one siderophore transporter system [16]; hence, a single mutation in or deletion of one transporter may not completely prevent siderophore-mediated influx. Consequently, in most cases, multiple mutations in different bacterial siderophore uptake transporters are required to effectively limit the entry of a siderophore-antibiotic conjugate [16]. More importantly, loss of function of *tonB* or *tonB3* (in *Acinetobacter* spp.), which encodes the primary proton motive force provider for active transport in Gram-negative bacteria, can completely retard bacterial growth in iron-depleted environments, such as occurs during an infection [12,17].

In this study, the novel siderophore cephalosporin, GT-1, exhibited potent activity against many *E. coli*, *K. pneumoniae* and *Acinetobacter* spp. MDR strains. However, high GT-1 MICs were observed for some strains. Non-susceptibility to GT-1 exhibited by some *K. pneumoniae* strains was often associated with the presence of AmpC β-lactamase DHA-1, for which the GT-1 MICs ranged from 4 to 64 μg/mL, with the exception of strain YMC2011/11/B1440. As we found no sequence differences over nearly 1000 bp upstream of the *bla*_DHA-1_ gene (data not shown), reduced *bla*_DHA-1_ expression seems an unlikely explanation; thus, the reason for the lower GT-1 MIC for this isolate remains to be determined. As expected, an increase in GT-1 MIC was observed in strain DH5α, which was transformed with ZpUC19::*bla*_DHA-1_, confirming the sensitivity of GT-1 to β-lactamase DHA-1.

In *Acinetobacter* spp. panel strains, very high GT-1 MICs were observed in strains harbouring *bla*_PER-1_, including YMC2003/01/R306, YMC2011/2/C582, YMC2003/5/C86 and YMC2013/3/R2081. The significant increase in GT-1 MIC (≥1024-fold, to 32 μg/mL) in *E. coli* DH5α transformed with ZpUC19::*bla*_PER-1_ supports the hypothesis that *bla*_PER-1_ contributes to higher GT-1 MICs in *Acinetobacter* spp. strains.

Another important aspect of our study was the systemic evaluation of siderophore uptake systems and porin loss in *E. coli* and *K. pneumoniae* panel strains and the resulting effect on GT-1 MICs. Previous reports demonstrated that single and double knock-out mutations of *cir* and *fiu* in *E. coli* decrease inhibition zones of catecholate siderophore-conjugated antibiotics [16]. However, our data suggest that GT-1 is efficiently transported into *E. coli* and *K. pneumoniae* cells with multiple defects in siderophore transporters. This could be explained by the structure of the GT-1 siderophore, which is a fusion of hydroxamate and catecholate that can be transported via hydroxamate or catecholate receptors. In addition, GT-1 did not exhibit a decrease in potency against isolates with single or double deletions of the porin genes *ompC* and/or *ompF* in *E. coli* or of *ompK35* and/or *ompK36* in *K. pneumoniae*. These results were in accordance with previous findings regarding siderophore-conjugated antibiotics [16] and suggest that the novel siderophore dihydroxypyridone is a suitable conjugant for the development of novel antibiotics. We observed similar findings for GT-1 with *Acinetobacter* spp. strains. In our analyses, GT-1 MICs were not high in double or triple *bauABCDE* mutants in strains YMC2012/09/R2209, YMC2012/01/R79, YMC2011/07/R812 and YMC2003/01/R306. This supports the hypothesis that GT-1 can be taken up via a transport system other than that involved in acinetobactin uptake and underscores the need for additional studies of GT-1 in this species.

Our study also demonstrated that GT-1 is active against *Acinetobacter* spp. AmpC-producing strains (CMY-2-, ADC-22, ADC-25, ADC-30, ADC-31, ADC-77), OXA-carbapenemase-producing strains (OXA-23, OXA-48, OXA-82, OXA-120, OXA-213, OXA-412, OXA-499), a serine carbapenemase-producing strain (KPC-2) and a metallo-carbapenemase-producing strain (IMP-1), with MICs ≤2 μg/mL. The significant potency of GT-1 against *Acinetobacter* spp. is one of its major strengths in comparison with CAZ-AVI, as CAZ-AVI is not approved for and exhibits minimal activity against *Acinetobacter* spp. Another important advantage is that GT-1 exhibits activity against IMP-1 metallo-β-lactamase–producing isolates, whereas avibactam is inactive against metallo-β-lactamase producers [18]. In view of the finding that CAZ-AVI resistance has emerged in KPC-producing *K. pneumoniae* with the L169P mutation in the Ω loop of KPC-2 and KPC-3 [19,20], GT-1 represents a potential alternative choice in the antimicrobial arsenal. An additional advantage we found is the high potency of the synergistic combination of GT-055 and GT-1, in which GT-055 enhanced GT-1 activity in the presence of β-lactamases in CTX-M- (CTX-M-14, CTX-M-15, CTX-M-27, CTX-M-55, CTX-M-65), SHV- (SHV-12, SHV-83), DHA-1- and SIM-1-producing strains. GT-055 exhibits intrinsic activity against many Enterobacteriaceae isolates, which likely contributes to its activity in combination with GT-1 against isolates of *E. coli* and *K. pneumoniae*. Recent studies have shown that GT-055 binds tightly to PBP2 in these species; thus, the synergistic activity of the combination likely reflects both the ability of GT-055 to inhibit β-lactamases as well as a direct bactericidal effect via PBP2-associated inhibition of cell wall biosynthesis [11]. The combination of GT-1/GT-055 exhibited better in vitro activity than AZT-AVI and CAZ-AVI, especially against *Acinetobacter* spp. panel strains.

## 4. Materials and Methods

### 4.1. Isolates

The clinical isolates examined in this study were obtained from a university-affiliated hospital in South Korea and collected from 2013 to 2017. For *E. coli*, approximately 2000 isolates were screened to select for specific antimicrobial-resistant phenotypes. *Acinetobacter* spp. and *K. pneumoniae* isolates were selected from our previously characterised panel strains [21,22]. Three *K. pneumoniae* strains (YMC2016/01/R859, YMC2016/02/N207 and YMC2016/04/N62) exhibiting carbapenem resistance were also added. Finally, 13 *E. coli* strains, 14 *K. pneumoniae* strains and 11 *Acinetobacter* spp. strains expressing different β-lactamases (narrow-spectrum and ESBL, KPC- and OXA-carbapenemases, metallo- and AmpC) were selected. MDR strains were defined as exhibiting resistance to at least three different antibiotic classes [23].

### 4.2. Test Compounds

Antibiotic agents included aztreonam (Dong-A Biotech Co., Seoul, Korea), ceftazidime (CJ Health Care, Seoul, Korea), meropenem (Yuhan Co., Seoul, Korea), imipenem (Choongwae Co., Seoul, Korea) and colistin (Sigma Aldrich, MO, USA). Avibactam was kindly provided by LegoChem Biosciences. GT-1 (or LCB10-0200) and GT-055 (or LCB18-055) were manufactured by LegoChem Biosciences.

### 4.3. Antimicrobial Susceptibility Tests

MICs for bacterial strains were determined using the Mueller–Hinton agar dilution technique, according to CLSI M07-A10 10th edition and M100 28th edition guidelines (2018) [24,25]. For colistin, interpretations were based on the European Committee on Antimicrobial Susceptibility Testing guidelines version 9.0 (2019). Antibiotic agents used as comparators included aztreonam, ceftazidime, meropenem, imipenem and colistin. The β-lactamase inhibitor avibactam was tested in combination with aztreonam and ceftazidime. GT-055 was included to assess the synergistic effect with GT-1. Antibiotic concentrations ranged from 0.12 to 256 μg/mL. GT-055 was tested at 4 μg/mL, similar to the avibactam concentration recommended by the CLSI guidelines. A previous study reported no change in GT-1 activity against cells grown on iron-depleted Muller–Hinton medium [26]. Therefore, the in vitro activity of GT-1 and GT-1/GT-055 under iron-depleted conditions was not assessed in this study.

### 4.4. DNA Extraction and Whole-Genome Sequencing

Bacteria were cultured overnight in Luria-Bertani broth at 37 °C. One millilitre of the overnight bacterial culture was used, and genomic DNA was extracted using a Wizard genomic DNA purification kit (Promega, WI, USA) according to the manufacturer’s instructions. Whole-genome library DNA was prepared using a Miseq reagent kit v3 and sequenced using Miseq v3.2 × 300-bp paired-end read cartridges (Illumina, CA, USA).

### 4.5. Sequence Assembly, Genome Annotation, MLST Determination and Resistome Analysis

Raw reads were assembled using SPAdes v3.11 [27]. Annotations were performed with the Rapid Annotation using Subsystem Technology pipeline [28]. Resistome data were collected using Resfinder v1.2 [29] and further verified using NCBI BLAST (http://blast.ncbi.nlm.nih.gov). Genomic analyses were performed using Geneious pro 8.1.9 (https://www.geneious.com). Bacterial sequence typing was conducted using the online MLST tool, 1.8 [30]. 

### 4.6. Cloning

Following PCR amplification with the primers listed in Table S1, *bla*_DHA-1_ with *Hin*dIII and *Eco*RI restriction site ends and *bla*_PER-1_ with *Bam*HI and *Eco*RI restriction site ends were cloned into ZpUC19. The ZpUC19 constructs were then transformed into *E. coli* DH5α, and GT-1 MICs were determined. Zeocin (50 μg/mL) was used for colony selection.

### 4.7. Analysis of Siderophore Uptake System and Porin Loss

Well-characterised siderophore transporters and porins from previous studies were included in the analysis (Table 4). The GenBank accession numbers of reference sequences are listed in Table S2. The selected target genes were mapped against the whole genome sequences of the panel strains using Bowtie alignment [31]. The mapped DNA sequences were translated into protein sequences to identify mutation-associated alterations affecting protein coding.

### 4.8. Accession Numbers

The draft whole-genome sequences of strains YMC2016/06/N138, YMC2016/07/N348, YMC2017/02/N55, YMC2017/01/N19, YMC2017/07/B11634, YMC2016/02/N176, YMC2016/06/N255, YMC2017/04/N120, YMC2017/06/P238, YMC2016/01/N95, YMC2016/01/C905, YMC2016/04/R3267, YMC2016/10/N189, YMC2016/01/R859, YMC2016/02/N207 and YMC2016/04/N62 were deposited in the NCBI database under the accession numbers SSKC00000000, SSKE00000000, SSJX00000000, VKOH00000000, SSKB00000000, SSJY00000000, SSKD00000000, VKOI00000000, SSKA00000000, SSJV00000000, SSJW00000000, SSJZ00000000, SSKF00000000, SSKG00000000, SSKH00000000 and SSKI00000000, respectively.

## 5. Conclusions

GT-1 exhibited MICs ≤2 μg/mL against many MDR isolates, including ESBL-, AmpC- and carbapenemase-producing *E. coli* and *K. pneumoniae* and OXA-producing *Acinetobacter* spp. In addition, GT-055 enhanced the in vitro activity of GT-1 against GT-1–resistant *E. coli*, *K. pneumoniae* and some isolates of *Acinetobacter* spp. Finally, DHA-1 and PER-1 increased GT-1 MICs against *K. pneumoniae* and *Acinetobacter* spp. strains, respectively.

## Figures and Tables

**Table 1:**
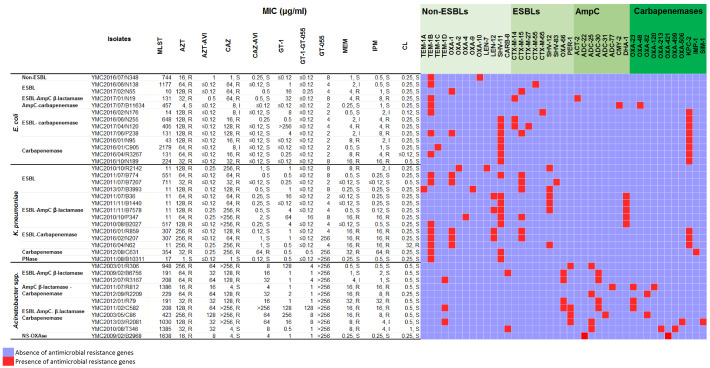
Antibiotic susceptibility and resistome of the *Escherichia coli*, *Klebsiella pneumoniae* and *Acinetobacter* spp. panel strains.

Abbreviations: AZT, aztreonam; AZT-AVI: aztreonam/avibactam; CAZ, ceftazidime; CAZ-AVI, ceftazidime/avibactam; MEM, meropenem; IPM, imipenem; CL: colistin, ESBL, extended-spectrum β-lactamase; Pnase, penicillinase; NS-OXAse, narrow-spectrum oxacillinase; MLST, multilocus sequence type; MIC, minimum inhibitory concentration. Antibiotic susceptibility testing was performed using the agar dilution method. Interpretation followed the Clinical and Laboratory Standards Institute guidelines M100, 28th ed., with the exception of colistin, in which the European Committee on Antimicrobial Susceptibility Testing (EUCAST) guidelines v9.0 were applied.

**Table 2:**
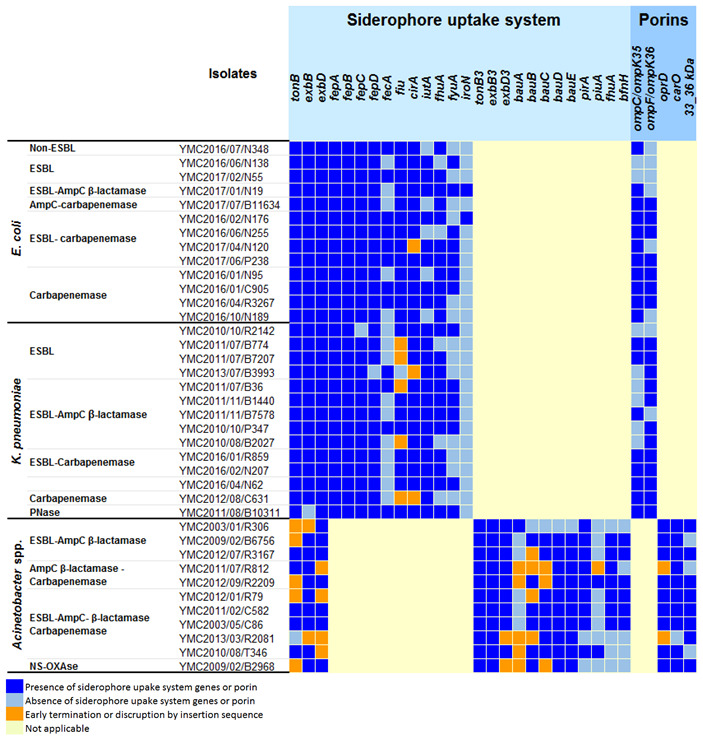
Porins and siderophore uptake systems of *E. coli*, *K. pneumoniae* and *Acinetobacter* spp. panel strains.

**Table 3:** Minimum inhibitory concentrations (MICs) of transformed *E. coli* DH5α strains.

Strain	GT-1 MIC (μg/mL)	Fold Change
DH5α+ ZpUC19::*bla*_DHA-1_	0.5	↑ 16-fold
DH5α+ ZpUC19::*bla*_PER-1_	32	↑ 1024-fold
DH5α+ ZpUC19	0.03125	−

↑: the arrow indicated the increase in fold change.

**Table 4:** Siderophore uptake transporters in *E. coli*, *K. pneumonia* and *A. baumannii*.

No.	Gene Code	Gene Name	Membrane Position	Reference
1	*tonB*	Ton complex subunit B	Inner membrane	[32]
2	*exbB*	Biopolymer transport subunit B	Inner membrane	[32]
3	*exbD*	Biopolymer transport subunit D	Inner membrane	[32]
4	*tonB3*	Ton complex subunit B	N/D	[12]
5	*exbB3*	Biopolymer transport subunit B3	N/D	[12]
6	*exbD3*	Biopolymer transport subunit D3	N/D	[12]
7	*fepA*	Ferric enterobactin outer membrane transporter	Outer membrane	[33]
8	*fepB*	Ferric enterobactin-binding periplasmic protein	Periplasm	[34]
9	*fepC*	Ferric enterobactin transport ATP-binding protein	Inner membrane	[35]
10	*fepD*	Ferric enterobactin transport system permease protein	Inner membrane	[36]
11	*fecA*	Ferric citrate outer membrane transporter	Inner membrane	[33]
12	*fiu*	Catecholate siderophore receptor	Outer membrane	[37]
13	*cirA*	Ferric dihyroxybenzoylserine outer membrane transporter	Outer membrane	[32]
14	*iutA*	Ferric aerobactin receptor	Outer membrane	[38]
15	*fhuA*	Ferrichrome outer membrane transporter receptor	Outer membrane	[33]
16	*bauA*	Ferric acinetobactin receptor	Outer membrane	[39]
17	*bauB*	Ferric acinetobactin transport system periplasmic binding protein	Inner membrane	[40]
18	*bauC*	ABC-type enterochelin transport system, permease component	Inner membrane	[41]
19	*bauD*	Ferric acinetobactin transport system permease	Inner membrane	[41]
20	*bauE*	ABC-type enterochelin transport system ATPase component	N/D	[42]
21	*pirA*	Ferric enterobactin receptor	Outer membrane	[43]
22	*piuA*	Hydroxamate-type ferrisiderophore receptor	Outer membrane	[43]
23	*iroN*	Salmochelin uptake receptor IroN	Outer membrane	[44]
24	*fyuA*	Yersiniabactin uptake receptor	Outer membrane	[45]
25	*bfnH*	Baumannoferrin uptake receptor	Outer membrane	[46]

N/D: Not determined.

## Supplementary Materials

The following are available online at https://www.mdpi.com/2079-6382/9/5/267/s1: Table S1: List of primers for cloning experiments, Table S2: GenBank accession numbers and reference gene loci used for alignment to siderophore uptake systems.
