# Supplementary material for: In Vitro Activity of a Novel Siderophore-Cephalosporin, GT-1 and Serine-Type β-Lactamase Inhibitor, GT-055, against Escherichia coli, Klebsiella pneumoniae and Acinetobacter spp. Panel Strains
Source: Antibiotics (Basel). 2020 May 20;9(5):267. doi: 10.3390/antibiotics9050267 (PMC7277296; doi:10.3390/antibiotics9050267)
Supplement: Supplementary file 1 [file antibiotics-09-00267-s001.pdf]

Supplementary materials

Table S1. List of primers for cloning experiments

Primer name	Sequences (5'→3')
DHA-1_Cloning_Fdw	CGAGAAGCTTGACCATAATCCACCTGTAAG
DHA-1_Cloning_Rev	CGCGAGAATTCTTATTCCAGTGCACTCAAAATAG
PER-1_Cloning_Fdw	CTATGGATCCTCGTTTTAGCCCTCTGGGCGTTC
PER-1_Cloning_Rev	CGCGAGAATTCTTAATTTGGGCTTAGGGCAG

Table S2. GenBank accession numbers and reference gene loci used for alignment to siderophore uptake systems

No.	Gene	GenBank accession number; locus tag		
		E. coli	*K. pneumoniae*	*A. baumannii*
1	*tonB*	U00096.3; b1252	CP009208.1; VK055_0270	CP000521.1; A1S_3708
2	*exbB*	U00096.3; b3006	CP009208.1; VK055_4048	CP000521.1; A1S_1605
3	*exbD*	U00096.3; b3005	CP009208.1; VK055_4049	CP000521.1; A1S_1604
4	*tonB3*	N/A	N/A	CP000521.1; A1S_0452
5	*exbB3*	N/A	N/A	CP000521.1; A1S_0453
6	*exbD3*	N/A	N/A	CP000521.1; A1S_0454
7	*fepA*	U00096.3; b0584	CP009208.1; VK055_1934	N/A
8	*fepB*	U00096.3; b0592	CP009208.1; VK055_1926	N/A
9	*fepC*	U00096.3; b0588	CP009208.1; VK055_1930	N/A
10	*fepD*	U00096.3; b0590	CP009208.1; VK055_1928	N/A
11	*fecA*	U00096.3; b4291	LR134202.1; NCTC9180_04189	N/A
12	*fiu*	U00096.3; b0805	CP009208.1; VK055_1207	N/A
13	*cirA*	U00096.3; b2155	CP009208.1; VK055_4930	N/A
14	*iutA*	CP032204.1; M340_11420	CP040175.1; FDZ08_15545	N/A
15	*fhuA*	U00096.3; b0150	CP009208.1;VK055_2400	N/A
16	*fyuA*	CU928163.2 ECUMN_2278	CP009208.1; VK055_5116	N/A
17	*iroN*	AF135597.1	CP009208.1; VK055_RS25840	N/A
18	*bauA*	N/A	N/A	CP000521.1; A1S_2385
19	*bauB*	N/A	N/A	CP000521.1; A1S_2386
20	*bauC*	N/A	N/A	CP000521.1; A1S_2388
21	*bauD*	N/A	N/A	CP000521.1; A1S_2389
22	*bauE*	N/A	N/A	CP000521.1; A1S_2387
23	*pirA*	N/A	N/A	CP000521.1; A1S_0980
24	*piuA*	N/A	N/A	CP000521.1; A1S_0474
25	*bfnH*	N/A	N/A	CP000521.1; A1S_1655

N/A: Not applicable
